# Burnout and coping mechanisms among Sudanese healthcare workers during the ongoing Sudan war: a cross-sectional study

**DOI:** 10.1186/s12995-025-00472-7

**Published:** 2025-07-31

**Authors:** Muhannad Bushra Masaad Ahmed, Ahmed Balla M. Ahmed, Salma Alrawa, Ludn Emad Ebrahim Mustafa, Mahmoud Elsadig Mahmoud Ali, Mohammed Osman Omer Abdalla, Sohaib Mohammed Mokhtar Ahmed

**Affiliations:** 1 Faculty of Medicine and Health Sciences, University of Gadarif, Gadarif, Sudan; 2 Faculty of Medicine, University of Khartoum, Khartoum, PO Box: 102, Al-Qasr Street, 11111 Sudan; 3 Faculty of Medicine, University of Bahri, Bahri, Sudan; 4 Faculty of Medicine and Health Sciences, University of Bakht Alruda, Eldewim, Sudan; 5 Faculty of Medicine and Health Sciences, University of Sennar, Sennar, Sudan

**Keywords:** Burnout, MBI-HSS, Healthcare workers, Coping mechanisms, Sudan, War

## Abstract

**Background:**

During the Sudan war, healthcare workers have encountered extraordinary challenges, including physical assaults and the immense strain of delivering care with critically limited resources. These conditions are likely to intensify burnout among healthcare professionals. This study aimed to determine the prevalence of burnout among Sudanese healthcare workers and investigate the coping mechanisms they employ during the ongoing conflict.

**Methods:**

A cross-sectional study was conducted among Sudanese healthcare workers using the standardized Maslach Burnout Inventory – Human Services Survey (MBI-HSS). Multivariable linear regression analysis was conducted to identify independent predictors of burnout domains. A significance level of *p* < 0.05 was considered for all statistical tests.

**Results:**

High emotional exhaustion was reported by 13.7% of participants, while 56.5% experienced high depersonalization, and only 4.4% reported low levels of personal accomplishment. The leading coping mechanism was talking with friends and family, adopted by 28.8% of participants, while 15.5% turned to spiritual or religious practices.

**Conclusion:**

Sudanese healthcare workers demonstrate high levels of burnout, particularly in the depersonalization domain, with talking to friends and family being the most commonly used coping mechanism. Future research is needed to explore the unique stressors faced by healthcare workers in conflict-affected settings like ours.

## Background

On April 15th, 2023, violent clashes erupted between the Sudanese Armed Forces (SAF) and the paramilitary Rapid Support Forces (RSF), plunging Sudan into a devastating conflict. This war has resulted in the displacement of over 12.5 million people, including internally displaced persons (IDPs), asylum seekers, and refugees [1]. The health infrastructure has been severely crippled, with the World Health Organization (WHO) reporting that 70% of public and private healthcare facilities in war-affected states were forced to close by the end of 2023 [2]. The violence has also directly targeted medical personnel, with more than 200 documented violations against healthcare workers, including the killing of 38 staff members. Incidents of kidnapping, assault, and intimidation have created an atmosphere of fear, leading to a critical shortage of healthcare workers as many have fled or refused to work due to safety concerns [3].

Living under wartime conditions, characterized by constant risk, uncertainty, and extreme suffering, exacerbates the loss of personal and professional resources. Such conditions are known to accelerate burnout processes in occupational settings and have long-term detrimental effects on mental health [4]. Burnout, a syndrome resulting from unmanaged chronic workplace stress, is characterized by three key dimensions: feelings of energy depletion or exhaustion, increased mental distance from one’s job (often manifested as negativism or cynicism), and reduced professional efficacy [5]. Among healthcare workers, burnout not only undermines individual well-being but also compromises the functioning of the entire healthcare system. It is associated with an increased risk of medical errors, reduced patient safety, and lower patient satisfaction, as well as higher rates of complaints from patients and their families [6].

Even prior to the current conflict, studies had already highlighted significant levels of burnout among Sudanese healthcare professionals. One study among resident physicians reported that 70.7% experienced high emotional exhaustion, while 44.2% showed high levels of depersonalization [7]. Likewise, during the COVID-19 pandemic, 45.7% of healthcare workers were found to be experiencing burnout [8]. These findings underscore the pre-existing vulnerabilities and psychological strain within Sudan’s healthcare workforce. Since the outbreak of the war, healthcare workers in Sudan have faced unprecedented challenges, including physical attacks, accusations of partisanship, and the overwhelming burden of providing care with severely limited resources in the few remaining functional hospitals [9]. These conditions are likely to exacerbate burnout and further deteriorate the mental health of healthcare workers, creating a vicious cycle that undermines both their well-being and the quality of care they can provide.

Studies from conflict-affected low- and middle-income countries have reported high levels of burnout among healthcare workers. For instance, a study among Palestinian health professionals during ongoing political violence found a burnout prevalence of 72.9%, with 44.2% experiencing emotional exhaustion and 9.8% reporting depersonalization [10]. However, no studies to date have assessed burnout among healthcare workers in Sudan during the current war. Therefore, this study aims to assess the prevalence of burnout among Sudanese healthcare workers and explore the coping mechanisms they employ to navigate these extraordinary circumstances.

## Methods

### Study design

This cross-sectional study was conducted in November 2024. It included healthcare workers from various healthcare facilities in the safest states of Sudan. The findings and methodology of this study were meticulously reported in the manuscript in accordance with the STROBE (Strengthening the Reporting of Observational Studies in Epidemiology) guidelines.

### Study population

The study included healthcare professionals, defined as individuals directly involved in patient care and healthcare delivery, such as physicians, nurses, technicians, pharmacists, and allied health staff, aged 18 years or older. Eligible participants had been employed at their current facility for at least one month to ensure they had adequate exposure to the work environment and stressors necessary for a meaningful assessment of burnout. Participation required written informed consent and completion of the study questionnaire. Exclusions included incomplete or inconsistent responses, those on long-term leave during data collection, and individuals previously diagnosed with mental health conditions such as anxiety or depression, as well as those receiving medications or therapy for any mental health issues.

### Sampling

We estimated the sample size using the Cochrane formula, given the unavailability of official population records of healthcare workers in Sudan. Assuming a population proportion of 50%, a margin of error of 5%, and a confidence interval of 95%, the minimum sample size required was calculated to be 385 participants. Ultimately, 563 healthcare workers participated in the study.

Convenience sampling was employed due to the challenges in accessing official healthcare workers records. Data collection was conducted through a combination of face-to-face interviews and an online survey administered via Google Forms. Trained medical students carried out the interviews in a confidential setting to promote honest and accurate responses while safeguarding participants’ privacy. They received prior instruction to ensure ethical conduct, accuracy, and consistency throughout the process. The online survey was shared among healthcare workers using widely used social media platforms in Sudan, such as WhatsApp, Telegram, and Facebook. Participation was entirely voluntary, anonymous, and without any form of compensation.

### Study instrument

The study employed a comprehensive tool comprising two sections, 13 questions in the socio-demographic section, covering age, sex, marital status, residence, displacement status, job roles, years of experience, exposure to conflict, living situation, access to basic necessities, access to mental health support, type of healthcare facility, and coping mechanisms. The second section of the questionnaire utilized the Maslach Burnout Inventory– Human Services Survey (MBI-HSS), which is the most widely accepted and commonly used tool for assessing burnout. It has been previously used in published research conducted in Sudan [7]. The MBI-HSS consists of 22 items divided into three domains: emotional exhaustion (6 items), depersonalization (8 items), and personal accomplishment (8 items) [11]. Each item was scored on a 7-point frequency scale (0 = Never to 6 = Every day). Emotional exhaustion scores were categorized as low (≤ 16), moderate [17–26], and high (≥ 27). Depersonalization scores were classified as low (≤ 6), moderate [7–12], and high (≥ 13). Personal accomplishment scores were categorized inversely, with low (≥ 39), moderate [32–38], and high (≤ 31) indicating higher levels of burnout. This study relied on the primary definition of burnout, as cited by previous studies [12, 13], which considers individuals with either high emotional exhaustion (≥ 27) or high depersonalization (≥ 13) as experiencing burnout. The MBI-HSS was administered in English, as it is the primary language used in Sudanese hospitals and among healthcare professionals [14].

### Statistical analysis

Data were analyzed using IBM SPSS Statistics, version 27. Descriptive statistics—means, standard deviations, frequencies, and percentages—were used to summarize demographic characteristics, burnout scores, and coping strategies. Multivariable linear regression analysis was conducted to identify independent predictors of burnout domains. All demographic and contextual variables identified a priori from existing literature (e.g., age, sex, job role, exposure to conflict, access to necessities) were simultaneously entered into the model. No variable selection procedures (e.g., stepwise regression) were applied. Significance was defined at *p* < 0.05 for all tests.

## Result

### Demographic factors

The study sample consisted of 563 participants, with a mean age of 27.9 years (SD = 6.3). The majority of the participants were female (61.1%), and most were single (77.8%). In terms of work experience, 43.5% had less than 1 year of experience and 4.1% had more than 15 years of experience. Regarding exposure to conflict situations, 19.5% reported no exposure, while 3.2% had extreme exposure. The majority of participants (70%) were living with family during the conflict with 16.2% lived alone. Access to basic necessities varied, with 23.1% having fully sufficient resources and 5.9% having extremely insufficient resources. 28.4% had access to psychological services, while 71.6% did not. In terms of healthcare facilities, most participants were affiliated with public hospitals (72.8%) while 21.2% worked in private hospitals. Finally, job roles varied, with physicians making up 64.3% of the participants, followed by technicians (10.5%) and pharmacists (10.1%) (Table 1).

**Table 1:** Socio-demographic characteristics

Variable	Overall (*N* = 563)	Variable	Overall (*N* = 563)
Age		Years of Work Experience	
Mean (SD)	27.9 (6.3)	Less than 1 year	245 (43.5%)
Sex		1–5 years	218 (38.7%)
Female	344 (61.1%)	6–10 years	58 (10.3%)
Male	219 (38.9%)	11–15 years	19 (3.4%)
Marital Status		More than 15 years	23 (4.1%)
Single	438 (77.8%)	Exposure to Conflict Situations	
Married	116 (20.6%)	None	110 (19.5%)
Divorced	6 (1.1%)	Minimal	148 (26.3%)
Widowed	3 (0.5%)	Moderate	193 (34.3%)
Residence		High	94 (16.7%)
Blue Nile State	3 (0.5%)	Extreme	18 (3.2%)
Darfour Region	1 (0.2%)	Living Situation during Conflict	
East Kordofan	1 (0.2%)	Alone	91 (16.2%)
Gadarif	143 (25.4%)	In shared accommodation with colleagues	78 (13.9%)
Gezira	25 (4.4%)	With family	394 (70.0%)
Kassala	49 (8.7%)	Access to Basic Necessities (Medical, Food, Supplies)	
Khartoum	36 (6.4%)	Fully sufficient	130 (23.1%)
North Kordofan	5 (0.9%)	Mostly sufficient	199 (35.3%)
Northern State	71 (12.6%)	Insufficient but manageable	201 (35.7%)
Red Sea State	58 (10.3%)	Extremely insufficient	33 (5.9%)
River Nile State	82 (14.6%)	Access to Mental Health Support	
Sennar State	21 (3.7%)	Yes (Psychologist, counselor, etc.)	160 (28.4%)
South Kordofan	1 (0.2%)	No	403 (71.6%)
White Nile State	67 (11.9%)	Type of Healthcare Facility	
Internally displaced persons (IDPs)		Public Hospital	409 (72.8%)
Yes	362 (64.3%)	Private Hospital	119 (21.2%)
No	201 (35.7%)	Community Pharmacy	11 (2.0%)
Job Role		Private Pharmacy	6 (1.1%)
Physician	362 (64.3%)	Non-governmental organization (NGO)/Relief Organization	19 (3.4%)
Technician	59 (10.5%)		
Pharmacist	57 (10.1%)		
Nurse	37 (6.6%)		
Medical laboratory	23 (4.1%)		
Administrative staff	14 (2.5%)		
Dentist	11 (2.0%)		

### Burnout

The burnout domains revealed varied experiences across healthcare workers. Emotional exhaustion was reported by 68.0% of participants as low, 18.3% as moderate, and 13.7% as high. Depersonalization had a higher percentage, with 56.5% of participants experiencing high depersonalization. Personal accomplishment was high for 90.9% of participants (Table 2).

**Table 2:** Burnout domains (MBI-HSS)

Burnout Domain	Mean (SD)	Range	Low	Moderate	High
**Emotional Exhaustion**	12.5 (10.4)	0.0–36.0	383 (68.0%)	103 (18.3%)	77 (13.7%)
**Depersonalization**	15.2 (11.5)	0.0–48.0	157 (27.9%)	88 (15.6%)	318 (56.5%)
**Personal Accomplishment**	11.7 (11.2)	0.0–48.0	25 (4.4%)	26 (4.6%)	512 (90.9%)

Regression analysis revealed several significant predictors across the three burnout dimensions. For emotional exhaustion, individuals with 1–5 years of work experience reported significantly higher scores compared to those with less than 1 year (β = 2.28, *p* = 0.026). Living in shared accommodation with colleagues during the conflict (β = 4.76, *p* = 0.003) and having insufficient but manageable access to basic necessities (β = 4.42, *p* < 0.001) were also associated with higher emotional exhaustion. Additionally, participants with extreme exposure to conflict had significantly elevated emotional exhaustion scores (β = 5.32, *p* = 0.043). Higher depersonalization scores were observed among participants with 1–5 years of work experience compared to those with less than 1 year (β = 2.35, *p* = 0.042), as well as among those with extreme conflict exposure (β = 8.26, *p* = 0.005) and insufficient but manageable access to basic necessities (β = 2.78, *p* = 0.043). Conversely, lower depersonalization scores were reported by those who had no access to mental health support (β = − 2.16, *p* = 0.048). For personal accomplishment, fewer predictors reached significance; however, participants reporting insufficient but manageable basic necessities had higher scores (β = 3.91, *p* = 0.004), and those with mostly sufficient access also showed slightly elevated scores (β = 2.62, *p* = 0.047). Other sociodemographic variables such as age, sex, marital status, and job role did not significantly predict burnout outcomes across the three domains (Table 3).

**Table 3:** Adjusted associations between demographic/contextual factors and MBI subscale scores (Multivariable linear regression)

Predictor	Emotional Exhaustion (β [95% CI], *p*)	Depersonalization (β [95% CI], *p*)	Personal Accomplishment (β [95% CI], *p*)
IDPs (Ref: No)			
Yes	1.41 [−0.40, 3.23], 0.127	–0.93 [–2.97, 1.11], 0.369	–0.82 [–2.83, 1.18], 0.421
Age	0.07 [–0.15, 0.28], 0.532	–0.05 [–0.29, 0.20], 0.711	–0.03 [–0.27, 0.20], 0.780
Sex (Ref: Female)			
Male	–0.83 [–2.63, 0.97], 0.367	1.09 [–0.93, 3.12], 0.290	–0.91 [–2.90, 1.09], 0.374
Marital Status (Ref: Single)			
Married	–0.21 [–2.64, 2.23], 0.869	–1.17 [–3.91, 1.57], 0.402	0.19 [–2.51, 2.89], 0.889
Divorced	6.35 [–2.00, 14.69], 0.136	5.43 [–3.95, 14.81], 0.256	2.89 [–6.35, 12.14], 0.539
Widowed	–2.15 [–14.34, 10.04], 0.729	–6.54 [–20.24, 7.17], 0.349	–5.46 [–18.96, 8.05], 0.428
Job Role (Ref: Physician)			
Technician	–3.40 [–6.21, − 0.60], **0.017**	–0.76 [–3.91, 2.39], 0.637	–1.84 [–4.94, 1.27], 0.246
Pharmacist	2.31 [–0.64, 5.26], 0.124	2.99 [–0.33, 6.30], 0.077	1.90 [–1.36, 5.17], 0.253
Nurse	–0.99 [–4.56, 2.58], 0.586	3.68 [–0.33, 7.69], 0.072	1.65 [–2.30, 5.60], 0.413
Lab Staff	–3.40 [–7.73, 0.93], 0.124	2.15 [–2.71, 7.02], 0.385	–1.62 [–6.41, 3.18], 0.508
Admin Staff	–0.96 [–6.40, 4.47], 0.727	5.64 [–0.47, 11.74], 0.070	4.15 [–1.87, 10.17], 0.176
Dentist	0.66 [–5.43, 6.75], 0.832	–2.54 [–9.39, 4.30], 0.466	0.63 [–6.11, 7.38], 0.854
Work Experience (Ref: <1 year)			
1–5 years	2.28 [0.27, 4.29], **0.026**	2.35 [0.09, 4.61], **0.042**	0.41 [–1.82, 2.63], 0.719
6–10 years	–0.06 [–3.46, 3.33], 0.971	1.22 [–2.60, 5.03], 0.532	0.98 [–2.78, 4.74], 0.609
11–15 years	0.82 [–4.75, 6.39], 0.772	–2.22 [–8.48, 4.04], 0.486	–3.21 [–9.38, 2.96], 0.308
> 15 years	–2.21 [–8.00, 3.57], 0.453	–0.41 [–6.92, 6.09], 0.901	0.27 [–6.14, 6.69], 0.933
Conflict Exposure (Ref: None)			
Minimal	1.43 [–1.11, 3.98], 0.269	1.40 [–1.46, 4.26], 0.338	0.72 [–2.10, 3.54], 0.615
Moderate	1.50 [–0.99, 3.99], 0.236	1.78 [–1.02, 4.57], 0.213	1.79 [–0.97, 4.54], 0.203
High	2.13 [–0.86, 5.11], 0.162	2.04 [–1.32, 5.39], 0.233	2.68 [–0.62, 5.99], 0.111
Extreme	5.32 [0.16, 10.49], **0.043**	8.26 [2.46, 14.06], **0.005**	4.67 [–1.05, 10.39], 0.109
Living Situation (Ref: Alone)			
Shared accommodation with colleagues	4.76 [1.68, 7.85], **0.003**	3.51 [0.04, 6.97], **0.047**	3.06 [–0.35, 6.48], 0.079
With family	0.69 [–1.69, 3.06], 0.569	1.96 [–0.71, 4.63], 0.150	2.39 [–0.24, 5.02], 0.075
Access to Basic Necessities (Ref: Fully sufficient)			
Mostly sufficient	3.12 [0.79, 5.46], **0.009**	1.82 [–0.80, 4.44], 0.173	2.62 [0.03, 5.20], **0.047**
Insufficient (manageable)	4.42 [2.03, 6.82], **< 0.001**	2.78 [0.09, 5.48], **0.043**	3.91 [1.25, 6.56], **0.004**
Extremely insufficient	2.70 [–1.22, 6.63], 0.176	3.77 [–0.64, 8.18], 0.093	3.70 [–0.64, 8.05], 0.095
Mental Health Support (Ref: Yes)			
No	0.46 [–1.44, 2.37], 0.633	–2.16 [–4.30, − 0.02], **0.048**	–0.35 [–2.46, 1.76], 0.746

Note: Bold values indicate statistically significant results (*p* < 0.05)

### Coping mechanisms

**Fig. 1:**
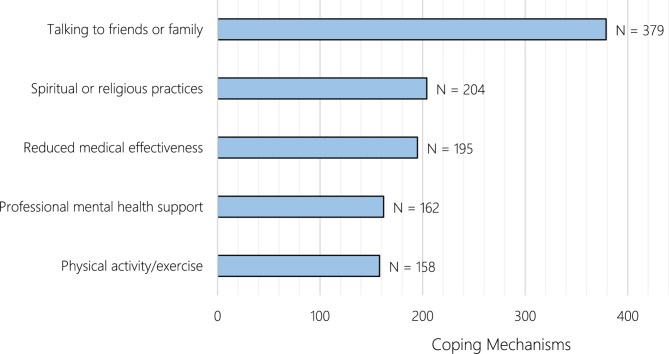
Coping mechanisms among participants

The coping mechanisms employed by participants in response to stress and challenges are diverse, as illustrated in Fig. 1. The most commonly used coping strategy is talking to friends or family, reported by 28.8% of participants. Other prominent methods include spiritual or religious practices (15.5%), which help individuals navigate difficult situations, and reduced medical effectiveness (14.8%), highlighting a coping method linked to compromised health care delivery. Professional mental health support is used by 12.3% of participants, while physical activity or exercise is another coping mechanism, favored by 12.0%.

## Discussion

Burnout among healthcare workers can impair clinical decision-making, hinder effective communication with patients and colleagues, and reduce their capacity to manage work-related stress [15]. This study aimed to assess the prevalence of burnout among Sudanese healthcare workers and explore the coping strategies they use amid the ongoing conflict.

The ongoing Sudanese military conflict has severely impacted healthcare services due to infrastructure destruction and a critical shortage of health workers [16]. The WHO has verified 88 attacks on healthcare resulting in 55 deaths and 104 injuries [17]. Incidents of killing, kidnapping, and assault have worsened the shortage of healthcare staff [3]. This is further exacerbated by shortage of medications and equipment [18, 19] and outbreaks of diseases like cholera and dengue [20, 21]. These factors likely contribute to the high levels of burnout among healthcare workers in Sudan. It has been suggested that there is an interaction between burnout and secondary traumatic stress in the Middle East [22].

Depersonalization was the most affected in our study, followed by emotional exhaustion and, finally, personal accomplishment, with only 4.4% reporting low levels of personal accomplishment. This aligns with findings from other countries [23, 24]. Notably, the proportion of participants experiencing depersonalization in our study was higher than that reported among healthcare workers in Gaza during their war [25]. These findings suggest that physicians in conflict-affected settings have a strong sense of self accomplishment, however factors like the workload, staff shortage, and working environment cause emotional exhaustion and depersonalization leading to high levels of burnout. Depersonalization and emotional exhaustion are considered the core components of burnout, whereas reduced personal accomplishment may act as either a precursor or an outcome of the syndrome [26]. The particularly high levels of depersonalization observed may indicate a psychological coping mechanism, whereby healthcare workers detach emotionally from their patients as a way to shield themselves from chronic stress [26]. In settings like Sudan, where prolonged trauma exposure and critical resource limitations are common, this emotional distancing can serve as a necessary—though potentially harmful—adaptation to survive in a highly strained and unsupportive healthcare environment.

Nearly two-thirds of the participants in this study were internally displaced. Sudan has the largest displacement crisis, more than 11 million internally displaced people [27]. There was no significant association between displacement status and burnout domains in this study. Less than one-fifth of participants had more than five years of experience, which may reflect the characteristics of healthcare practitioners remaining in Sudan during the war. Senior physicians are more likely to find jobs abroad to support their families, while younger physicians, who lack training, are more likely to stay. This pattern may also reflect differences in social media use and availability, as younger physicians are more likely to complete online questionnaires. These plausible explanations require further investigations to obtain robust evidence. Burnout levels varied by years of experience, with early-career professionals (1–5 years) showing significantly higher emotional exhaustion and depersonalization. This finding is consistent with previous studies that have reported greater burnout among those with fewer years of experience [28, 29].

Nurses were more likely to report depersonalization compared to physicians; however, this difference was not statistically significant. A study conducted among healthcare workers during the COVID-19 pandemic found that Sudanese physicians were more likely to report burnout compared to nurses. A plausible explanation for this finding is the heavier workload and frontline role of physicians during COVID-19. This contrasts with findings from other studies that report higher levels of burnout among nurses [30, 31]. Some studies, however, report no significant difference between the two groups [32]. Technicians were significantly less likely to experience emotional exhaustion compared to physicians, a difference that may also be attributed to workload.

Less than a quarter of participants reported having fully sufficient access to basic necessities. This is consistent with the general situation in war-torn Sudan, where one in every three people is at risk of food insecurity [33]. Insufficient but manageable access to basic necessities was significantly associated with high levels of emotional exhaustion and depersonalization. A probable explanation is that the affected access domain is financial, which can be managed by working longer hours in multiple hospitals. This year, food prices have increased by more than 120% [34].

More than two-thirds of participants reported a lack of access to psychological support. The expertise of mental health and psychological support groups is fragmented, and there is no shared platform to coordinate priority actions or strengthen responses to mental health needs [35].

Participants with extreme conflict exposure showed significantly higher levels of emotional exhaustion and depersonalization. Similarly, a study conducted among academic staff in Ukraine found that prolonged stress related to the ongoing war led to notable increases in depersonalization [36]. Despite the severe impact of the war, many conflict-related variables in our study were not significantly associated with burnout in our adjusted models. One possible explanation is that the conflict affected nearly all healthcare workers in some way, creating a uniformly high baseline of stress and reducing detectable differences across exposure levels. Additionally, the effects of the war may have manifested more through indirect stressors, such as economic hardship, longer working hours, and lack of basic resources, which were better captured by other variables like access to necessities and living arrangements.

The most commonly reported coping mechanisms were talking to friends and families as well as spiritual or religious practices. Previous studies have found that social relationships may help in managing the effects of burnout [37, 38]. Additionally, religious practices are regarded as protective and beneficial in managing burnout [39, 40]. A previous study conducted among displaced individuals in shelters in Port Sudan highlighted the significant role of religious and spiritual practices—such as prayer, reading the Quran, and trusting in God—in coping with distress. The study also emphasized the importance of social and emotional support, noting that individuals often leaned on family ties and built supportive social relationships to manage their circumstances [41]. These coping strategies may reflect the strong communal and religious values embedded in Sudanese culture, where individuals often rely on close-knit family networks and faith during times of crisis. In the context of limited access to formal mental health services during the conflict, these culturally rooted mechanisms may serve as essential substitutes, helping individuals manage stress and emotional exhaustion.

This study offers valuable insights into burnout among healthcare providers in a conflict-affected country. A notable strength is the inclusion of participants from various professional roles within the healthcare system. However, the interpretation of the findings should consider several limitations. The cross-sectional design restricts the ability to draw causal inferences. Moreover, the use of a non-probability sampling technique may limit the generalizability of the results. Participants’ responses may have been influenced by recall bias and the tendency to provide socially desirable answers. Additionally, the disproportionate distribution of participants across healthcare roles—with physicians comprising approximately two-thirds of the sample—may affect the representativeness and limit the generalizability of the findings to the broader population of healthcare workers.

## Conclusion

Healthcare workers in Sudan exhibit a high level of burnout mainly in the depersonalization domain. However, the most commonly used coping mechanisms include talking to friends and family, as well as engaging in spiritual or religious practices. There is a need for context-specific research into the unique stressors faced by healthcare workers in conflict-affected settings like ours. In addition, healthcare workers should receive targeted training on evidence-based strategies to manage and prevent burnout.

## Data Availability

“The datasets used and/or analyzed during the current study are available from the corresponding author on reasonable request.”

## Abbreviations

MBI-HSS: Maslach Burnout Inventory– Human Services Survey
SAF: Sudanese Armed Forces
RSF: Rapid Support Forces
IDPs: Internally displaced persons
WHO: World Health Organization
STROBE: Strengthening the Reporting of Observational Studies in Epidemiology
NGO: Non-governmental organization

## References

[1] UNHCR. Sudan Crisis Explained. UNHCR. 2024. Available from: https://www.unrefugees.org/news/sudan-crisis-explained/.

[2] Elamin A, Abdullah S, Abda ElAbbadi A, Abdellah, Hakim A, Naiema Wagiallah et al. Sudan: from a forgotten war to an abandoned healthcare system. BMJ Global Health. 2024;9(10):e016406–6. Available from: https://gh.bmj.com/content/9/10/e016406. Cited 2024 Nov 4. 10.1136/bmjgh-2024-016406 PMC11529772 39477337

[3] Badri R, Dawood I. The implications of the Sudan war on healthcare workers and facilities: a health system tragedy. Conflict Health. 2024. 10.1186/s13031-024-00581-w. 38494471 10.1186/s13031-024-00581-w PMC10946115

[4] Hakhmigari MK, Diamant I. Occupational burnout during war: the role of stress, disruptions in routine, sleep, work-family conflict, and organizational support as a moderator. PLoS One. 2025;20(1):e0316917-7. 39854439 10.1371/journal.pone.0316917 PMC11759394

[5] World Health Organization. Burn-out an. Occupational Phenomenon: International Classification of Diseases. World Health Organization. 2019. Available from: https://www.who.int/news/item/28-05-2019-burn-out-an-occupational-phenomenon-international-classification-of-diseases.

[6] Izdebski Z, Kozakiewicz A, Białorudzki M, Dec-Pietrowska J, Mazur J. Occupational Burnout in Healthcare Workers, Stress and Other Symptoms of Work Overload during the COVID-19 Pandemic in Poland. Int J Environ Res Public Health. 2023;20(3):2428 Available from: https://www.ncbi.nlm.nih.gov/pmc/articles/PMC9916221/. 36767797 10.3390/ijerph20032428 PMC9916221

[7] Elhadi YAM, Ahmed A, Salih EB, Abdelhamed OS, Ahmed MHH, El Dabbah NA. A cross-sectional survey of burnout in a sample of resident physicians in Sudan. Kirgiz MS, editor. Plos One. 2022;17(3):e0265098. 10.1371/journal.pone.0265098 PMC8896711 35245338

[8] Esraa SA, Alfadul, Idrees M, Alrawa SS, Osman RO, Hassan H, Alsamany T, Albasheir et al. Burnout and its associated factors among healthcare workers in COVID-19 isolation centres in Khartoum, Sudan: A cross-sectional study. Plos One. 2023;18(7):e0288638–8. Available from: https://www.ncbi.nlm.nih.gov/pmc/articles/PMC10361487/. Cited 2023 Oct 4. 10.1371/journal.pone.0288638 PMC10361487 37478101

[9] Dalouk K, Haar RJ. Protecting health in conflict in Sudan: a call for health worker solidarity. BMJ. 2023;381:p1453. Available from: https://www.google.com/url?q=https://www.bmj.com/content/381/bmj.p1453%26;sa=D%26;source=docs%26;ust=1707794362193077%26;usg=AOvVaw2FWD7q1Wa1LoM0tVbIr0G. Cited 2024 Feb 13. 10.1136/bmj.p1453 37369388

[10] Ahmead M, El Sharif N, Alwawi A, Hemeid A, Ziqan M. The prevalence of burnout and coping strategies among Palestinian health professionals: a cross sectional study. Frontiers in Public Health. 2024;12. Available from: https://pmc.ncbi.nlm.nih.gov/articles/PMC11688348/. 10.3389/fpubh.2024.1477812 PMC11688348 39744357

[11] Erwan F, Iqbal M, Hasanuddin I, Zuhri S, Maydini CM. Burnout syndrome analysis among hospital nurses using Maslach Burnout Inventory - Human Service Survey (MBI-HSS): a case study. IOP Conference Series: Materials Science and Engineering. 2020;931: 012025.

[12] Rotenstein LS, Torre M, Ramos MA, Rosales RC, Guille C, Sen S, et al. Prevalence of Burnout Among Physicians. JAMA. 2018;320(11):1131 Available from: https://jamanetwork.com/journals/jama/fullarticle/2702871. 30326495 10.1001/jama.2018.12777 PMC6233645

[13] Li-Sauerwine S, Rebillot K, Melamed M, Addo N, Lin M. A 2-Question summative score correlates with the Maslach burnout inventory. WestJEM 213 May Issue. 2020;21(3):610–7. 10.5811/westjem.2020.2.45139 PMC7234685 32421508

[14] Ali Dinar G. E. English as a Medium of Instruction in Arab-Speaking Hospitals: Opportunities and Challenges. Egyptian J Hospital Med. 2025;99(1):2319–25. Available from: https://ejhm.journals.ekb.eg/article_432811.html?utm_source=chatgpt.com. Cited 2025 Jul 2.

[15] Batanda I. Prevalence of burnout among healthcare professionals: a survey at fort portal regional referral hospital. npj Mental Health Res. 2024;3(1):1–10. Available from: https://www.nature.com/articles/s44184-024-00061-2#:~:text=Burnout%20among%20healthcare%20professionals%20can. 10.1038/s44184-024-00061-2 PMC11074248 38710834

[16] Dafallah A, Osman, Ibrahim M, Rania E, Elsheikh, Blanchet K. Destruction, disruption and disaster: sudan’s health system amidst armed conflict. Confl Health. 2023;17(1):43. 10.1186/s13031-023-00542-9 PMC10523736 37752590

[17] WHO condemns the increasing attacks on health care amid Sudan’s war. World Health Organization - Regional Office for the Eastern Mediterranean. 2024. Available from: https://www.emro.who.int/sdn/sudan-news/who-condemns-the-increasing-attacks-on-health-care-amid-sudans-war.html.

[18] Sudan Restrictions and lack of medicines deprive people in Khartoum state of lifesaving care| MSF. Médecins Sans Frontières (MSF) International. 2023. Available from: https://www.msf.org/sudan-restrictions-and-lack-medicines-deprive-people-khartoum-state-lifesaving-care. Cited 2025 Mar 1.

[19] Soaring medical needs mark 500 days of war in Sudan| MSF. Médecins Sans Frontières (MSF) International. 2024. Available from: https://www.msf.org/soaring-medical-needs-mark-500-days-war-sudan.

[20] UNICEF. Over Three Million Children at Heighted Risk of Cholera and other Deadly Diseases in Sudan. Unicef.org. 2024. Available from: https://www.unicef.org/sudan/press-releases/over-three-million-children-heighted-risk-cholera-and-other-deadly-diseases-sudan.

[21] First famine, now cholera and dengue fever surge hits war-torn Sudan, UN News. 2024. Available from: https://news.un.org/en/story/2024/11/1156461.

[22] Chemali Z, Ezzeddine FL, Gelaye B, Dossett ML, Salameh J, Bizri M, et al. Burnout among healthcare providers in the complex environment of the Middle East: a systematic review. BMC Public Health. 2019. 10.1186/s12889-019-7713-1. 31640650 10.1186/s12889-019-7713-1 PMC6805482

[23] Magalhães E, Oliveira ÁCM, de Govêia S, Ladeira CS, Queiroz LCA, Vieira DM. Prevalence of burnout syndrome among anesthesiologists in the federal district. Brazilian J Anesthesiology. 2015;65(2):104–10. 10.1016/j.bjan.2013.07.016 25740276

[24] Ugwu FO, Ugwu C, Njemanze VC, Nwosu I. Family cohesion and family size moderating burnout and recovery connection. Occup Med. 2018;69(1):28–34. 10.1093/occmed/kqy155 30476256

[25] Aldabbour B, Dardas LA, Hamed L, Alagha H, Alsaiqali R, El-shanti N, et al. Emotional exhaustion, depersonalization, and personal accomplishment: exploring burnout in Gaza’s healthcare workforce during the war. Middle East Curr Psychiatry. 2025. 10.1186/s43045-025-00519-9.

[26] Edú-Valsania S, Laguía A, Moriano JA, Burnout. A review of theory and measurement. International Journal of Environmental Research and Public Health. 2022;19(3):1–27. Available from: https://pmc.ncbi.nlm.nih.gov/articles/PMC8834764/. 10.3390/ijerph19031780 PMC8834764 35162802

[27] Displacement in Sudan Crosses 11 Million as Devastating Crisis Reaches New Heights: IOM Chief. International Organization for Migration. 2021. Available from: https://www.iom.int/news/displacement-sudan-crosses-11-million-devastating-crisis-reaches-new-heights-iom-chief.

[28] Youssef D, Youssef J, Abou-Abbas L, Kawtharani M, Hassan H. Prevalence and correlates of burnout among physicians in a developing country facing multi-layered crises: a cross-sectional study. Sci Rep. 2022;12(1): 12615. 35871153 10.1038/s41598-022-16095-5 PMC9308770

[29] Patel R, Bachu R, Adikey A, Malik M, Shah M. Factors Related to Physician Burnout and Its Consequences: A Review. Behav Sci. 2018;8(11). Available from: https://www.ncbi.nlm.nih.gov/pmc/articles/PMC6262585/. 10.3390/bs8110098 PMC6262585 30366419

[30] Giselle Dayana Valdes-Elizondo, Álvarez-Maldonado P, Maria Angélica Ocampo-Ocampo, Hernández-Ríos G, Réding-Bernal A, Hernández-Solís A. Burnout symptoms among physicians and nurses before, during and after COVID-19 care. Revista Latino-americana De Enfermagem. 2023;31:e4046. 10.1590/1518-8345.6820.4047 PMC10631294 37937599

[31] Dubale BW, Friedman LE, Chemali Z, Denninger JW, Mehta DH, Alem A, et al. Systematic review of burnout among healthcare providers in sub-Saharan Africa. BMC Public Health. 2019. 10.1186/s12889-019-7566-7. 31510975 10.1186/s12889-019-7566-7 PMC6737653

[32] Papazian L, Hraiech S, Loundou A, Herridge MS, Boyer L. High-level burnout in physicians and nurses working in adult ICUs: a systematic review and meta-analysis. Intensive Care Med. 2023;400. 10.1007/s00134-023-07025-8. 10.1007/s00134-023-07025-8 PMC10041519 36971792

[33] Sudan, Summary of the Humanitarian Needs and Response Plan and the Regional Refugee Response Plan (February. 2024)| OCHA. www.unocha.org.2024. Available from: https://www.unocha.org/publications/report/sudan/sudan-summary-humanitarian-needs-and-response-plan-and-regional-refugee-response-plan-february-2024.

[34] Sudan war. Nearly 26 million going hungry due to rising food prices, access challenges| UN News. news.un.org. 2024. Available from: https://news.un.org/en/story/2024/07/1152426.

[35] Mental Health and Psychosocial Support (MHPSS) Technical Working Group in Sudan Terms of Reference (TOR.) - Sudan. ReliefWeb. 2024. Available from: https://reliefweb.int/report/sudan/mental-health-and-psychosocial-support-mhpss-technical-working-group-sudan-terms-reference-tor. Cited 2025 Mar 1.

[36] Tsybuliak N, Yana Suchikova, Shevchenko L, Popova A, Serhii, Kovachev. Olha Hurenko. Burnout dynamic among Ukrainian academic staff during the war. Scientific Reports. 2023;13(1). Available from: https://www.nature.com/articles/s41598-023-45229-6?utm_source=chatgpt.comCited 2025 Feb 22. 10.1038/s41598-023-45229-6 PMC10589323 37864023

[37] Doolittle BR. Association of burnout with emotional coping strategies, friendship, and institutional support among internal medicine physicians. J Clin Psychol Med Settings. 2020;15(2):361–7. 10.1007/s10880-020-09724-6 PMC7225246 32415546

[38] Rogers E, Polonijo AN, Carpiano RM. Getting by with a little help from friends and colleagues: Testing how residents’ social support networks affect loneliness and burnout. Canadian Family Physician. 2016;62(11):e677. Available from: https://pmc.ncbi.nlm.nih.gov/articles/PMC9844583/. PMC9844583 28661887

[39] De Diego-Cordero R, Iglesias-Romo M, Badanta B, Lucchetti G, Vega-Escaño J. Burnout and spirituality among nurses: A scoping review. Explore. 2021;18(5):612–20. 10.1016/j.explore.2021.08.001 34429263

[40] Doolittle BR, Windish DM, Seelig CB. Burnout, coping, and spirituality among internal medicine resident physicians. J Graduate Med Educ. 2013;5(2):257–61. 10.4300/JGME-D-12-00136.1 PMC3693690 24404269

[41] Alfahal MS, Bashar Alasha, Essam A, Tsabeh Abdelrahem Higazy, Khalid EM, Saif D et al. Conflict and displacement in Sudan: Health challenges, socio-economic strain, trauma, and coping mechanisms among internally displaced persons in Port Sudan shelters. Medicine. 2025;104(16):e42232–2. Available from: https://journals.lww.com/md-journal/fulltext/2025/04180/conflict_and_displacement_in_sudan__health.8.aspx?context=latestarticles#. 10.1097/MD.0000000000042232 PMC12014049 40258722

